# *TP53* transcription factor for the *NEDD9/HEF1/Cas-L* gene: potential targets in Non-Small Cell Lung Cancer treatment

**DOI:** 10.1038/srep10356

**Published:** 2015-05-26

**Authors:** Bénédicte ROUSSEAU, Catherine JACQUOT, Julie LE PALABE, Marine MALLETER, Christophe TOMASONI, Tifenn BOUTARD, Vehary SAKANYAN, Christos ROUSSAKIS

**Affiliations:** 1 IICiMED/EA 1155 - Département Cancer du Poumon et Cibles Moléculaires, UFR Sciences Pharmaceutiques - 9 rue Bias - BP53508 - 44035 NANTES CEDEX 1 - FRANCE; 2 UMR INSERM U 1085 Groupe “Death receptors and tumor escape” - Université de Rennes 1 Campus Santé - Bâtiment 5 - 2 avenue du Prof Léon Bernard - 35043 RENNES – France; 3 IICiMED/EA 1155 - Département Cancer du Poumon et Cibles Moléculaires, UFR Sciences Pharmaceutiques - 9 rue Bias - BP53508-44035 NANTES CEDEX 1–FRANCE

## Abstract

Lung cancer is a serious public health problem. Although there has been significant progress in chemotherapy, non-small cell lung cancer is still resistant to current treatments, primarily because of the slow rate of cell development. It is thus important to find new molecules directed against targets other than proliferation agents. Considering the high proportion of mutant proteins in tumor cells, and the high rate of mutation of the *TP53* gene in all cancers, and in NSCLC in particular, this gene is a perfect target. Certain new molecules have been shown to restore the activity of mutated p53 protein, for example PRIMA-1, which reactivates the His273 mutant p53. In a previous study, we presented triazine A190, a molecule with a cytostatic activity that blocks cells in the G1 phase and induces apoptosis. Here, we show that A190 not only restores mutant p53 activity, but also induces an overexpression of the *NEDD9* gene, leading to apoptotic death. These findings might offer hope for the development of new targeted therapies, specific to tumor cells, which spare healthy cells.

Regular progress is being made in chemotherapy, including for non-small cell lung cancer (NSCLC), which shows significant improvement when treated with cis-platin in association with older, conventional, molecules. Nevertheless NSCLC remains a major public health problem and remission for this type of cancer is rare, while the response rate to treatment and the five-year survival rate are both low^1^,^2^.

Thus there is a need to develop new strategies to treat NSCLC. As knowledge about the carcinogenesis process increases, treatment strategies are becoming more directed, for example, strategies targeting oncogenes that are overexpressed in NSCLC, such as Ras, Myc, Bcl2 and EGFR; or inactivated tumor suppressor genes like Rb and *TP53, i.e.* strategies that involve inhibiting oncogenes or reactivating tumor suppressor genes^3^.

This is the goal of the therapy designed to target the *TP53* gene. The high mutation rate of *TP53* in NSCLC makes it an ideal target in the development of new strategies. *TP53* is mutated in 50% of human cancers and in more than 70% of lung cancers^4^. These mutations cause the loss of function of the wild type protein p53, which normally plays a major role in tumor suppression. Certain hot spots, such as residue 273, are more affected than others. A number of studies have shown that a long inactivation of *TP53* is needed for significant tumor growth and that the restoration of *TP53* function can stop this growth and induce tumor regression^5^.

In recent years, various reactivation strategies have been proposed which try to replace the mutated gene or to modulate the functions of the protein. However, all have encountered problems, such as difficulties in administration or secondary effects on the wild type *TP53*^6^. An approach specifically targeting, through pharmacomodulation, only the mutated form of the protein would seem apposite as it would not affect healthy cells, which contain a wild type form of the protein.

At present, only a few molecules in development have the above-mentioned properties^7^,^8^. One of these is the PRIMA-1 molecule (P53 Reactivation and Induction of Massive Apoptosis)^9^. Developed by a Swedish laboratory, it has a significant cytostatic effect on cell lines with a mutated *TP53*, but is not toxic in wild type TP53 gene cell lines, as it has the capacity to restore the wild type spatial conformation of the central domain of mutated p53 proteins, or to create a new p53-DNA interaction. These proteins then increase their binding DNA capacity and in particular their effectiveness as a transcription factor. The mutated p53 can induce all the standard gene targets and reactivate their tumor suppressor role. Cell proliferation ceases and the cells die through apoptosis or senescence, depending on the cell line^10^. PRIMA-1 seems particularly active on cell lines with a mutation on the DNA-binding domain of p53 protein, primarily the His273 p53 mutant^11^. It also exhibits antitumor activity *in vivo* in SCID mice xenografted with Saos-2-His273 cells^12^. A derivate of PRIMA-1, APR-246, is currently in phase I of clinical trials^13^.

In a similar vein, our laboratory is developing a new molecule, A190, which can restore the transcription factor activity of mutated p53 proteins in an NSCLC cell line. Like PRIMA-1, A190 exhibits *in vivo* antitumor activity without apparent toxicity. In a previous work, we reported tumor regression on nude mice xenografted with NSCLC-N6-L16. In addition, A190, which is also cytostatic, induces blocking of the cell cycle in the G1 phase and apoptosis^14^. We showed that none of this activity was a result of the activation of the wild type *TP53* gene. In fact, the NSCLC-N6-L16 cell line carries the His273 p53 mutant, therefore the protein is inactive, and the mutant does not induce p21 expression, a major factor for G1 blocking^15^.

The reactivation of mutant p53 by A190 causes an original cell reaction and induces the expression of a new target: *NEDD9/HEF1/Cas L*, a gene discovered in 1996 by Golemis, which is involved in numerous cellular functions, including migration, mitosis, dsfferentiation and apoptosis^16^,^17^.

In the present paper, we provide strong evidence that, in the presence of A190, the His273 p53 mutated protein recovers its capacity to bind DNA and enables the overexpression of *NEDD9,* which leads to apoptosis. We demonstrate that *NEDD9* is a target gene for the His273 p53 mutated protein reactivated by treatment, which thus becomes a transcription factor for the *NEDD9* gene.

## Results

### Measurement of kinetic expression of the NEDD9 gene in synchronized NSCLC-N6-L16 cells

All qPCR analyses were carried out on synchronized cells. As the expression of *NEDD9* varies over the cell cycle^18^ it is necessary to study cells that are in the same cell cycle phase in order to measure accurately the effect of A190 on the expression of *NEDD9*.

**Figure 1:**
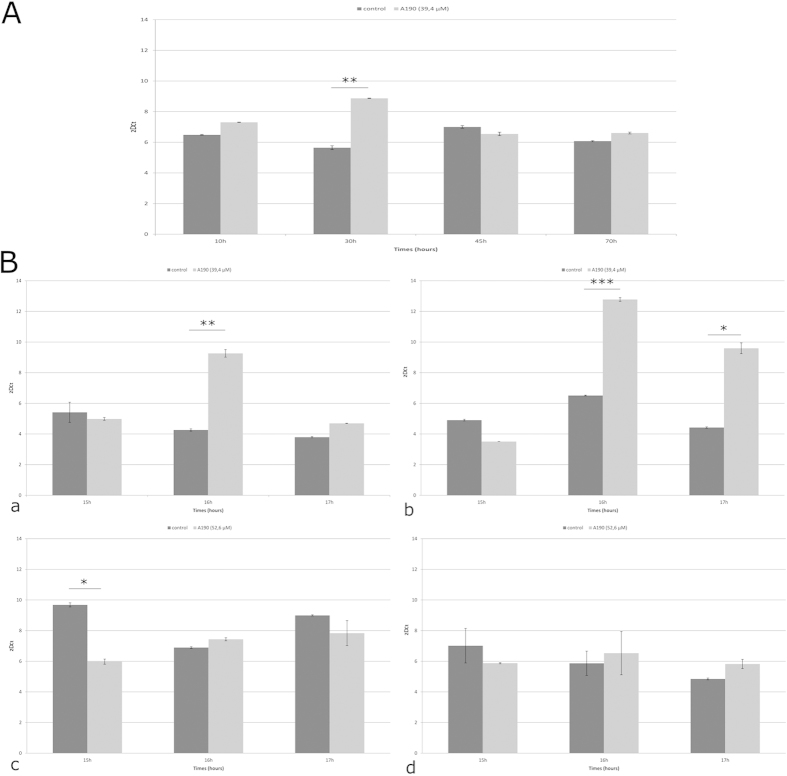
(**A**) Expression of *NEDD9* tested by qRT-PCR on synchronized NSCLC-N6-L16 cells treated or not with A190 at IC50 at 10, 15, 30 and 45 hours. (**B**) Expression of *NEDD9* (**a** and **c**) or *TP53* (**b** and **d**) tested by qRT-PCR on synchronized NSCLC-N6-L16 cells (**a** and **b**) or synchronized A549 cells (**c** and **d**) treated or not with A190 at IC50 from 15 to 17 hours. The results are expressed as a ratio of mRNA quantity of the genes tested and of β-actin (control gene). The values are mean ± S.D. (*n* = 8 for each group). (* p < 0.002 - ** p < 0.001 - ***p < 0.0002).

An initial kinetic study was carried out. Synchronized NSCLC-N6-L16 cells were combined with A190 from 0 to 70 hours. Every 5 hours, RNA messengers were extracted and the expression of *NEDD9* was quantified by qPCR. The results enabled the most interesting time points to be identified for a second kinetic study (data not shown). As a result, several time points were selected: 10 hours, 15 hours, 30 hours and 45 hours. (Fig. 1A) For all points, the expression of *NEDD9* was higher in treated NSCLC-N6-L16 cells than in control cells. However, for one time point the difference was greater: after 15 hours of treatment, significant overexpression was visible in comparison with the control group.

### Measurement of the comparative kinetic expression of NEDD9 and TP53 genes in synchronized NSCLC-N6-L16 cells

Based on the above-mentioned results (Fig. 1A), a third, more precise, study was, performed after about 15 hours of treatment in order to identify precisely the timing of the *NEDD9* overexpression and to compare the expression of *NEDD9* and *TP53*, an important factor in apoptosis. This was carried out on both NSCLC-N6-L16 and A549 cell lines treated with A190 at IC50 (Fig. 1B).

A190 induced significant overexpression of both *NEDD9* and *TP53* genes in synchronized NSCLC-N6-L16 cells; in fact, the expression in treated cells doubled for both genes compared to the control. This overexpression was exactly the same after 16 hours of treatment with A190 (Fig. 1B). Although the overexpression of *NEDD9* was only visible after 16 hours, *TP53* overexpression was still evident after 17 hours of treatment.

In contrast, in the A549 cell line, the expression of *NEDD9* remained low for the treated cells in comparison with control cells at almost all time points. However, the basal expression in non-treated cells was higher for A549 than for NSCLC-N6-L16. For all A549 samples treated with A190 there was no significant difference in the expression of *TP53* (Fig. 1B).

### Flow cytometry analysis of the effect of PRIMA-1 and A190 on the cell cycle

All histograms were obtained from synchronized NSCLC-N6-L16 or A549 cells in mitosis, treated or not with PRIMA-1 or A190 at IC50 for 16 hours. The IC50 for PRIMA-1 for both cell lines was identified in our laboratory: the values were 27 μM for NSCLC-N6-L16 and 34 μM for A549. First, the entire population of cells was examined, then the state of apoptosis of the cells was investigated.

### NSCLC-N6-L16 cell line

**Figure 2:**
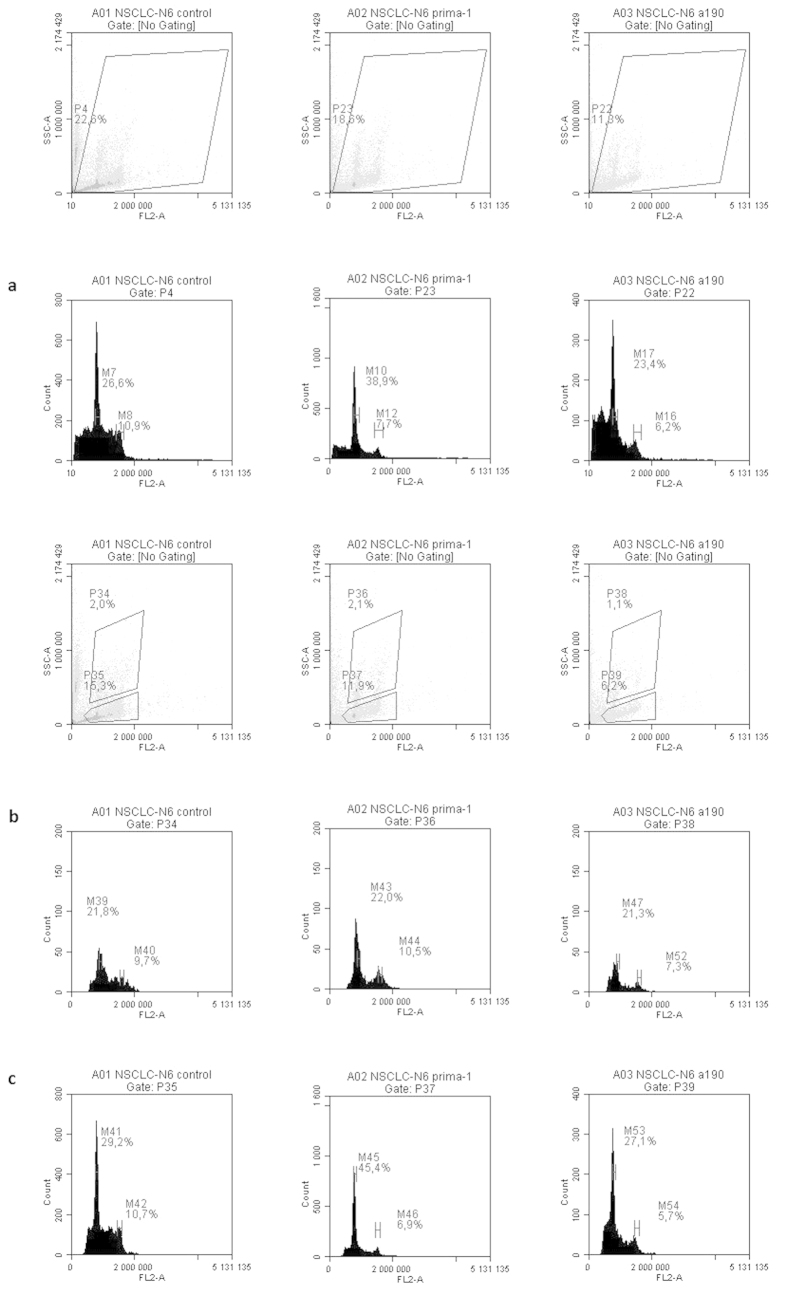
Flow cytometry analysis of the comparative effects of different cytostatic molecules on synchronized NSCLC-N6-L16 cells treated at IC50 for 16 hours (**a**: total cells **b**: non-apoptotic cells **c**: apoptotic cells).

Cells were synchronized 16 hours beforehand, during mitosis, thus the control-cell histogram shows cells essentially in the G1 phase of the cell cycle. However, some cells appeared in the S phase in the entire population study (Fig. 2A). The successive analyses of cells according to their state of apoptosis showed that this S phase was not real but corresponded to cell debris from cells that had died through apoptosis. On the histograms for non-apoptotic cells (Fig. 2B), only blocking in the G1 phase is visible. NSCLC-N6-L16 is a polyploid cell line^19^, which is why there are cells with either 2n DNA or 4n DNA in the G1 phase.

This false S phase in the control cells was caused by a large amount of cell debris due to an over-high density of cells in the wells and the mortality of the cells by apoptosis through lack of nutrients.

When treated with PRIMA-1, the proportion of cells in the G1 phase increased for both cells with 2n or 4n DNA. The amount of cell debris was lower as was the cell density compared to the control (Fig. 2).

A190 had a more powerful effect than PRIMA-1. Blocking in the G1 phase of the cell was still visible but only a few cells were countable on the histograms: only 27.1% of the apoptotic population remained in the G1 phase, whereas with PRIMA-1 there was still 45.4% (29.2% for the control). This shows that most cells had already suffered an apoptotic death: A190 does have a blocking effect but can also induce the destruction of cells by apoptosis (Fig. 2C).

### A549 cell line

**Figure 3:**
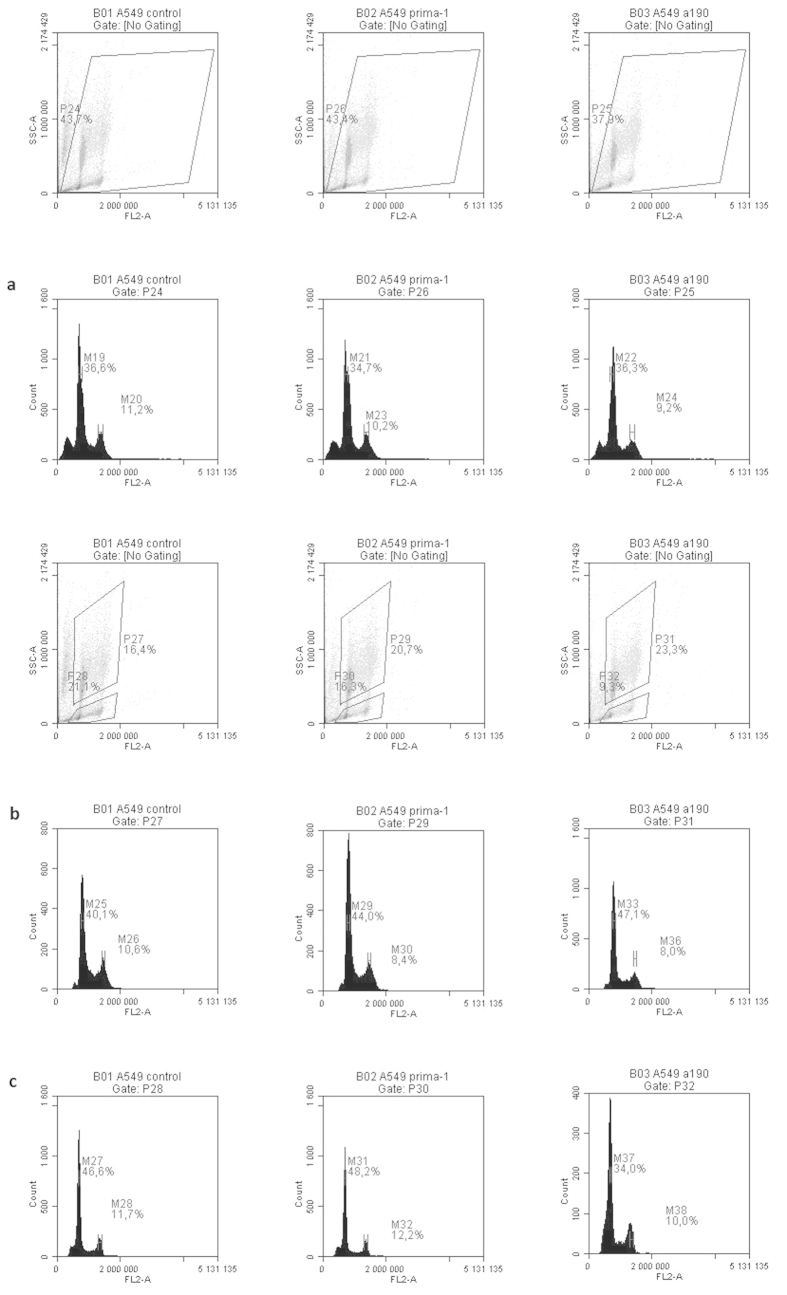
Flow cytometry analysis of the comparative effects of different cytostatic molecules on synchronized A549 cells treated at IC50 for 16 hours (**a**: total cells **b**: non-apoptotic cells **c**: apoptotic cells).

The false S phase was also apparent for the A549 cell line, even more so because of the faster doubling time of A549 compared to NSCLC-N6-L16 (Fig. 3).

As for the NSCLC-N6-L16 cell line, there was blocking in the 2n DNA population in the G1 phase of A549 cells when treated with either PRIMA-1 (44% of cells were in the G1 phase against 40.1% for the control) or A190 (47.1% against 40.1% for the control). However, the effect on the 4n DNA population was less significant than on the NSCLC-N6-L16 cell line for both treatments.

The difference between the two molecules was again visible in the apoptotic population. While blocking in the G1 phase was still higher in cells treated with PRIMA-1 than in control cells (48% against 46.6%), the proportion of cells blocked in the G1 phase was significantly lower for cells exposed to A190 (only 34%). This shows the greater efficacy of the molecule: the cells already being dead through apoptosis in this case.

### Restoration of the fixation of His273 p53 mutated protein to DNA after treatment with A190

**Figure 4:**
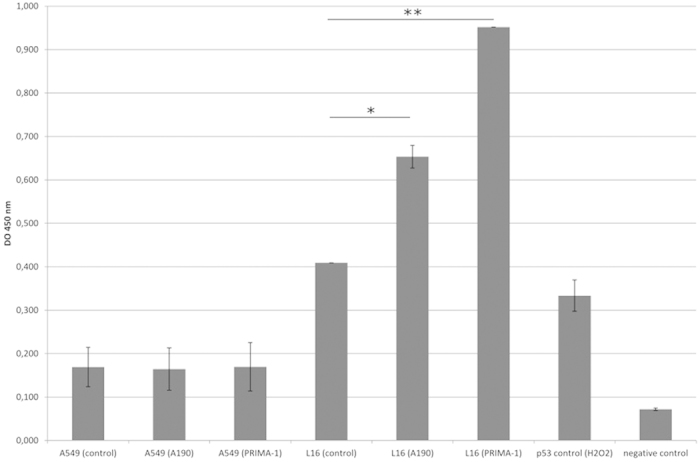
Observation of the degree of fixation of p53 to DNA in NSCLC-N6-L16 or A549 cells treated or not with PRIMA-1 or A190 at IC50. The values are mean ± S.D. from triplicate samples. (* p < 0.006 - ** p < 0.0001).

This assay was carried out with the Active Motif kit TransAM p53 in 96-well plates in which an oligonucleotide containing a p53 consensus binding site had been immobilized. It was performed with both cell lines treated with A190 molecule and PRIMA-1 at IC50 (Fig. 4).

An already relatively high fixation of the p53 protein on the consensus sequence could be observed in control cells. However, when treated with PRIMA-1, known for its capacity to restore the wild type conformation of His273 mutated p53, the fixation of the protein in NSCLC-N6-L16 cells increased dramatically by a factor of 2.5. Similarly, when treated with A190, the fixation of His273 mutated p53 protein increased considerably by a factor of 1.6.

In contrast, in the A549 cell line, while the fixation of wild type p53 proteins was observed for the control cells, treatment with PRIMA-1 or A190 did not modify the degree of binding of the protein.

### ChIP assays to identify links between the genome (NEDD9) and the proteome (p53): transcription factor-DNA binding interactions

In order to demonstrate a definitive link between p53 activation and *NEDD9* expression, a cross-linked chromatin from NSCLC-N6-L16 cells treated for 16 hours with A190 was prepared. This chromatin was then immunoprecipitated (IP) with antibody specific to p53. Finally, the IP genomic DNA was analyzed to measure the amount of *NEDD9* DNA using PCR with primers specific to the gene. A 10% total input sample of cross-linked chromatin was kept from every test as a control.

**Figure 5:**
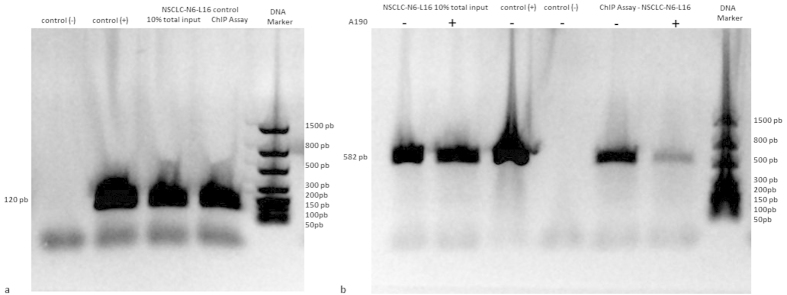
(**a**) ChIP assay control on synchronized NSCLC-N6-L16 with Anti-RNA Polymerase II Antibody and ChIP Positive Control Primers (GAPDH promoter) (**b**) ChIP assays on synchronized NSCLC-N6-L16 treated or not with A190 with p53 antibody and *NEDD9* specific primers.

A positive control was carried out with NSCLC-N6-L16 cross-linked chromatin immunoprecipitated with Anti-RNA Polymerase II antibody and the IP genomic DNA was analyzed using PCR with primers specific to the GAPDH promoter close to the transcription start site. In these controls, a 120 bp fragment was found, which corresponded to a positive control for the assay technique and reagent integrity (Fig. 5A).

For the 10% total input sample, a 582 bp fragment was found, which confirmed the presence of *NEDD9* in the cross-linked chromatin of each test. The presence of the same fragment in the NSCLC-N6-L16 cell ChIP assays demonstrated a physical link between *NEDD9* and p53 (Fig. 5B).

## Discussion

There are 37,000 new cases of lung cancer per year in France, with a survival rate below 14% after 5 years (http://www.e-cancer.fr). In order to develop new therapeutic strategies, it seems necessary to create new molecules specifically targeting the altered genes in tumor cells, thus sparing healthy cells. This would also enable the development of more personalized treatments for patients, whose altered genes are not systematically the same. The *TP53* gene is a very interesting target as, in abnormal cells, mutated p53 proteins lose their “guardian of the genome” function, responsible for halting proliferation and inducing apoptosis. In addition, some mutations can lead these denatured proteins gaining function, becoming oncoproteins. They then promote cell proliferation, accumulation of mutations in the cells and the tumor transformation process^20^.

*P53* is one of the most frequently mutated genes in cancer cells and particularly in lung cancer cells, more than 70% of which have a *TP53* mutation. The discovery of new treatments for these cancers has naturally turned to molecules able to target mutated p53 protein. In addition, His273 is a hot spot that is most frequently mutated in lung cancer cells^21^.

PRIMA-1 is one of the rare examples of molecules in development that can restore the activity of His273 p53 mutant proteins by restoring their wild type conformation^9^. The mutant p53 can then induce some classic gene targets of p53. For example, genes involved in cell cycle arrest (p21, GADD45 and 14-3-3d) or apoptosis (Bax, Noxa) can be expressed again in tumor cells. Cell proliferation is halted and the cells die by apoptosis or senescence, depending on the cell line^10^.

Our research into A190 ties in with this interest in targeted therapy. In previous work, we showed that A190 has *in vivo* antitumor properties without apparent toxicity. *In vitro* it exhibits irreversible cytostatic activity on both cell lines: NSCLC-N6-L16 and A549. This activity is linked to the blocking of the cell line in the G1 phase followed by apoptosis^14^. A further study established the His273 mutation by G->A transition on the *p53* gene in the NSCLC-N6-L16 cell line^15^. Finally, we demonstrated that apoptosis induced by A190 treatment in both cell lines is provoked by overexpression of *NEDD9*, specifically in the G1 phase of the cell cycle^14^.

*NEDD9* is involved in many cell processes and its role in apoptosis induction is particularly well documented. It is a ubiquitous gene strongly expressed in lung and lung cell lines^22^. Its overexpression is associated with many types of cancer and it seems to be a spread factor in the development of metastases^23^. However, it has been demonstrated that overexpression beyond a certain threshold is a pro-apoptotic factor. To promote proliferation, *NEDD9* must be at a precise level in cells; if it is overexpressed, or silenced by siRNA, it leads to apoptosis^24^,^17^.

The aim of the present study was to evaluate whether, like PRIMA-1, A190 can reactivate His273 p53 mutated protein and, at the same time, to investigate further the link between this reactivation of p53 and the overexpression of the *NEDD9* gene.

In NSCLC-N6-L16 cells, we have shown, by quantitative PCR, an overexpression of the *NEDD9* gene after 16 h of treatment with A190. In the same samples of RNA extract, an overexpression of p53 was observed at the same time point: 16 h. In the same experiment with an A549 cell line, no significant difference in the expression of *NEDD9* or *TP53* could be noted. However, the expression in non-treated A549 cells was higher than in non-treated NSCLC-N6-L16.

It has been previously reported that A549 cell lines lack *LKB1* and this absence presumably contributes to the upregulation of *NEDD9*^25^,^26^.Testing for the presence of *LKB1* in NSCLC-N6-L16 cells showed the expression of the gene in the cell line (data not shown), implying that the overexpression of *NEDD9* after 16 hours can only be a consequence of the treatment with A190. The fact that there was no significant modification of *NEDD9* gene expression with or without treatment in A549 cells proves the specificity of the A190 effect to cell lines with a mutated *TP53*.

The cytostatic and pro-apoptotic effects of A190 and PRIMA-1 on the cell cycle of both cell lines, NSCLC-N6-L16 and A549, were first examined and compared using flow cytometry analyses.

Apoptotic cells that still contain apoptotic bodies can be detected using flow cytometry: this is called the sub G1 peak^27^,^28^. This peak occurs just before G1 and is made up of cells that have not yet discharged their apoptotic bodies. Two populations of cells were differentiated in our analysis: an apoptotic population and a non-apoptotic population.

Analysis of both these populations in A549 showed that both PRIMA-1 and A190 could block cells in the G1 phase of the cell cycle. A190 had a higher pro-apoptotic capacity than PRIMA-1. The sub G1 apoptotic population was smaller after treatment with A190 than with PRIMA-1, which means that, when treated with A190, the cells were already dead and there were fewer cells still containing the apoptotic bodies.

Analysis of NSCLC-N6-L16 also showed a blocking in the G1 phase of the cell cycle induced by both PRIMA-1 and A190 in both populations of cells: apoptotic and non-apoptotic. In fact, the higher pro-apoptotic effect of A190 compared to that of PRIMA-1 was even more pronounced in this cell line: there were fewer cells in the sub G1 peak, which means that apoptotic bodies were released into the medium and were therefore no longer visible.

Treatment with either molecule induces blocking in the G1 phase of the cell cycle in both cell lines: NSCLC-N6-L16, which has a mutated *TP53*, and A549, which has a wild type *TP53*. As overexpression of *TP53* was only seen in NSCLC-N6-L16, it seems likely that the blocking in G1 and the apoptosis brought about by both pro-apoptotic treatments do not occur by the same signaling pathway.

With these results in mind, we then investigated whether A190 also reactivates His273 p53 mutated protein. With A549 cells, no modification in p53 protein fixation on DNA was revealed with A190 or with PRIMA-1. This follows from the fact that A549 has the wild type copy of the protein which is in its normal conformation and cannot be affected by the treatment. In NSCLC-N6-L16 cells, p53 proteins are mutated to His273, but they seem to retain their ability to fix to DNA, having a partially wild type conformation. In addition, mutant proteins accumulate in cells, which explains the already high proportion of fixation in control cells^29^. Above all, this test demonstrates the ability of A190 not only to restore the fixation of His273 p53 mutants to DNA but also to intensify this fixation. PRIMA-1 also exhibits this ability, related to its transactivation capability^30^. Thus, like PRIMA-1, which has a derivate in the clinical trial phase, A190 can restore the functions of p53.

Finally, we demonstrated a physical link between *NEDD9* and the p53 protein, enabling us to identify *NEDD9* as a new target for the transcription factor p53.

In conclusion, the present paper shows not only that the A190 molecule can restore the functions of His273 mutated p53 protein but also that, once reactivated, it can induce overexpression of *NEDD9,* which is controlled by the transcription factor function of p53 whose gene, *TP53*, is also overexpressed. This overexpression is specific to mutated proteins since only A190 induces the joint overexpression of *TP53* and *NEDD9* in cell lines including the mutation. The absence of overexpression of *NEDD9* in the A549 cell line, with a wild type *TP53* gene, coupled with the absence of induction of *TP53* confirms this specific activity to His273 mutated p53 protein. Finally, A190 only provokes a blockage in the G1 phase of the cell cycle and apoptotic death in the NSCLC-N6-L16 cell line when p53 is mutated. The overexpression of *NEDD9* in tumor cells, a new target gene of reactivated His273 p53 mutated protein, then leads cells to apoptosis.

This apoptosis of the cancer cells can be explained in two ways: either mutated p53 proteins, reactivated by treatment, induce the overexpression of *NEDD9* in the G1 phase of the cell cycle, instead of the G2/M phase, and this disrupts the normal cell cycle; or the levels of NEDD9 protein increase and cross the threshold. In both cases, the cell recognizes this as a trigger factor for apoptosis.

In this paper, we have demonstrated that the treatment of a non-small cell lung cancer cell line using the A190 molecule leads to the specific reactivation of His273 mutated p53 proteins. These can then bind to DNA again and play a transcription factor role for *NEDD9* in tumor cells.

An interesting subject for future research would be to investigate whether, like PRIMA-1, the A190 molecule induces the intrinsic pathway of apoptosis. To this end, we are currently carrying out qPCR analyses on the Bax and Bcl2 genes. We would like to confirm these results *in vivo* on nude mice xenografted with the NSCLC-N6-L16 cell line, in which we have already demonstrated the antitumor activity of the A190 molecule.

The development of such molecules, able to reactivate a tumor suppressor gene like *TP53*, which is mutated in more than 50% of cancer cases, and which can also act on a target like *NEDD9*, which is particularly involved in the carcinogenesis process, offers hope for the development of new targeted therapies. These therapies would then enable a very specific action on tumor cells while sparing healthy cells in the body.

## Materials and Methods

### Cell lines and cultures

Two cell lines, A549 and NSCLC-N6, were used in this study originating from an adenocarcinoma and an epidermoid lung cancer, respectively. NSCLC-N6 is a cell line derived from an NSCLC of a previously untreated patient (moderately differentiated classified as T2N0M0)^19^. The A549 line was obtained from the ATCC (reference CCL-185)^31^ and is known to have a wild type p53 gene, while NSCLC-N6 has a mutant p53 gene, similar to tumors *in situ*.

The cell lines were cultured in RPMI-1640 enriched with 100 IU penicillin, 100 μg/ml streptomycin, 2 mM glutamine and 5% fetal bovine serum. Cell culture plates were maintained in humidified incubators at 37 °C in a 5% CO_2_ atmosphere. NSCLC-N6 has a cell doubling time of 48 h *in vitro*, and A549 24 h.

### Cell synchronization in the M phase

M phase synchronization was chosen as all synchronization methods using biochemical agents can cause genotypic cell modifications plus a double cytotoxicity from the molecules and from the treatment studied and so may cause bias.

During mitosis, cell shape and adhesion change dramatically. Mitosis induces a depolymerization of the microtubules^32^,^33^ and a loss of the cytoskeleton and adhesion to the support. Thus the cells lie at the bottom of the culture flask throughout mitosis and it is easy to isolate them by slightly stirring the culture medium, taking the cells out and suspending them. The cells are then salvaged by slow centrifugation and placed in a new culture flask.

### A190

**Figure 6:**
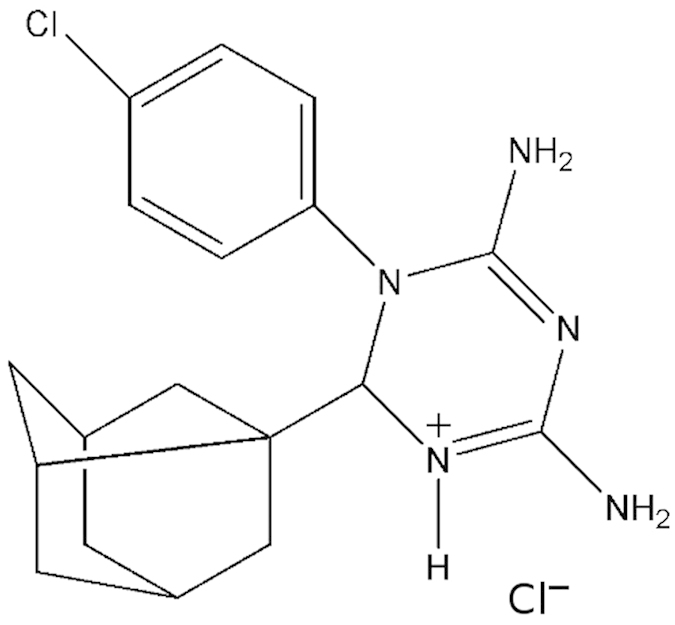
Chemical structure of the triazine A190.

A190 (4,6-diamino-1,2-dihydro-1-(400-chlorophenyl)-2-(1-tricyclo[3.3.1.13,4] decyl-1,3,5-triazine) is a product of chemical synthesis (Fig. 6). It was selected from more than 200 analogs using pharmacological assays. It is under international patent (Patent PCT: GR02/00036). The pure compound is dried and can be dissolved in water. The IC50 for A190 is 39.4 μM in NSCLC-N6 and 52.6 μM in A549.

### Extraction of total RNA

Total RNA was extracted from NSCLC-N6 and A549 cell lines using the Dynabeads® mRNA Direct^TM^ Kit (Ambion by Life Technologies). The isolation protocol relies on base pairing between the polyA residues at the 3’ end of most mRNA and the oligo (dT) residues covalently coupled to the surface of the Dynabeads®. The quality and concentration of purified RNA were assessed using UV absorbance at 260/280 nm and samples were run on 1% agarose gel in order to assess their quality. RNA was stored at −80 °C.

### cDNA synthesis

Reverse transcription (RT) was carried out with 1 μg of RNA in 10 μl with 0.125 μg/μl random primers and RNase-free water. This mixture was denatured at 65 °C for 10 min then kept on ice. Subsequently, it was completed to a final volume of 25 μl with a mix containing 8 mM of dNTP mix, 1X of M-MLV 5X reaction buffer, 25 units of recombinant RNasin ribonuclease inhibitor (Promega), and 200 units of M-MLV RT (Promega) and RNase-free water. In order to check whether the samples were contaminated by genomic DNA, the same mix was made with RNA without the reverse transcriptase. Following this step, samples were incubated for 2 h at 37 °C. cDNAs were stored at -20 °C. After reverse transcription, cDNAs were used as a template for specific qPCR.

### Quantitative real-time RT-PCR

Real-time RT-PCR was carried out in a final volume of 25 μl with 1 μl 1:20 dilution of diluted cDNA mixture, 10 pmol gene-specific forward and 10 pmol reverse primer in 1X SYBR Green PCR master mix (Eurogentec) with the following protocol: 15 sec at 95 °C for denaturation, 30 sec at 60 °C for annealing and extension on an ABI Prism Sequence Detection System 5700 (Applied Biosystems). The primers were designed using Primer 3 (http://frodo.wi.mit.edu/). All primer sequences were controlled with a dimmer finder to reduce the risk of unspecific hybridization of SYBR-green (https://www.operon.com/oligos/toolkit.php).

The primer sequences were *NEDD9*: forward 5’-CGCTGCCGAAATGAATAT-3’, reverse 5’-CCCTGTGTTCTGCTCTATGACG-3’ (10 mM, Sigma Genosys); *TP53*: forward 5’-GTTCCGAGAGCTGAATGAGG-3’, reverse 5’-TCTGAGTCAGGCCCT TCTGT-3’ (10 mM, Sigma Genosys). The relative expression of each gene was normalized to that of human β-actin: forward 5’-ATTCCCTTGCCTTCTTGGAT-3’, reverse 5’-CGTGAGGTCTGCCACTACAG-3’ (10 mM, Sigma Genosys). Normalization was carried out using the ΔΔCt method (Supplementary Figure). A non-template control sample was used for each PCR to check the genomic DNA contamination of the cDNA template. The results were analyzed using GenAmp 5700 SDS (Applied Biosystems) software.

### Cell cycle analysis

For DNA staining, 200 10^3^ cells were cultured in 9.4-cm^3^ Petri dishes in the absence and presence of A190. DNA staining was carried out using Vindelov’s technique^34^. The solution (0.01 M glycine/NaOH; 0.96 mM propidium iodide ; 0.1 M Nonidet P40; 700 IU ribonuclease A/I; 0.3 M NaCl; diluted 1:2 (v/v) in PBS) was put into flasks, which were then shaken and left in a dark environment at 4 °C for 15 min. The cell suspension thus obtained was filtered on nylon mesh (50 lm) and analyzed. The DNA content of at least 30,000 nuclei was measured on an Accuri C6 FACScan.

### Sequence-specific DNA binding of His273 p53

To quantify p53 activation a nuclear extract was made using a Nuclear Extract Kit (Active Motif). Protein was quantified using a ProStain™ Protein Quantification Kit (Active Motif) and the activation of p53 was studied using the Trans AM^TM^ p53 Transcription Factor Assay Kit (Active Motif). The latter contained a 96-well plate with an immobilized oligonucleotide containing a p53 consensus binding site (5‘-GGACATGCCCGGGCATGTCC-3’). p53 in the nuclear extract binds specifically to this oligonucleotide. The primary antibody used in the TransAM p53 Kit recognizes an accessible epitope on p53 protein upon DNA binding. Addition of a secondary HRP-conjugated antibody provides a sensitive colorimetric readout easily quantified by spectrophotometry.

### Chromatin Immunoprecipitation (ChIP)

After treatment, cells were fixed in a 1% formaldehyde solution for 15 min at room temperature. Glycine was added for an additional 5 min at room temperature to neutralize the formaldehyde. Cells were then pelleted at 3000 xg for 5 minutes at room temperature. The ChIP assays were carried out using a Thermo Scientific Pierce Agarose ChIP Kit. The following antibodies were used: p53 Antibody (PAb 240) (ThermoFisher Scientific; MA5-15244), Anti-RNA Polymerase II antibody and Normal Rabbit IgG (both included in the kit).

To amplify the HEF1 gene, the following primer sequences were used: forward 5’-AATCATGGGGACGGGTCTTT-3’, reverse 5’-TGACTAAGCCTGAAGTGGGG-3’ (10 mM, Sigma Genosys). PCR was carried out in a final volume of 50 μl with 18 μl of purified DNA mixture, 10 μl of 5X buffer (Promega), 8 μl of MgCl_2_ (Promega), 1 μl of dNTP 10 mM mix (Promega), 0.28 μl of GoTaq® Hot Start Polymerase 500 u (Promega) and 1.78 μl of each primer (1:10). PCR products were then detected using the FlashGel® System by Lonza.

## Additional Information

**How to cite this article**: ROUSSEAU, B. *et al.* *TP53* transcription factor for the *NEDD9/HEF1/Cas-L* gene: potential targets in Non-Small Cell Lung Cancer treatment. *Sci. Rep.* **5**, 10356; doi: 10.1038/srep10356 (2015).

## Supplementary Material

Supporting Information
