# Supplementary material for: TP53 transcription factor for the NEDD9/HEF1/Cas-L gene: potential targets in Non-Small Cell Lung Cancer treatment
Source: Sci Rep. 2015 May 26;5:10356. doi: 10.1038/srep10356 (PMC4443767; doi:10.1038/srep10356)
Supplement: Supporting Information [file srep10356-s1.pdf]

TP53 transcription factor for the *NEDD9/HEF1/Cas-L* gene: potential targets in Non-Small Cell Lung Cancer treatment

Bénédicte ROUSSEAU[1], Catherine JACQUOT[1], Julie LE PALABE[1], Marine MALLETER[2], Christophe TOMASONI[1], Tifenn BOUTARD[1], Vehary SAKANYAN[1], Christos ROUSSAKIS[3]*

[1]IICiMED/EA 1155 - Département Cancer du Poumon et Cibles Moléculaires, UFR Sciences Pharmaceutiques - 9 rue Bias - BP53508 - 44035 NANTES CEDEX 1 - FRANCE
[2]UMR INSERM U 1085 Groupe "Death receptors and tumor escape" - Université de Rennes 1 Campus Santé - Bâtiment 5 - 2 avenue du Prof Léon Bernard - 35043 RENNES - FRANCE
[3]IICiMED/EA 1155 - Département Cancer du Poumon et Cibles Moléculaires, UFR Sciences Pharmaceutiques - 9 rue Bias - BP53508 - 44035 NANTES CEDEX 1 – FRANCE - tel. +33 643 115 298 - fax. +33 253 484 150 - christos.roussakis@univ-nantes.fr

	10h control	10h A190	15h control	15h A190	30h control	30h A190	45h control	45h A190
Ct β-actin	15.8666	15.144	15.2566	15.1377	16.0344	15.7954	15.4569	16.1563
Ct β-actin	15.7546	15.1752	15.6013	15.114	16.0033	15.8294	15.4671	16.163
	**average**	**15.622**		**standard**	**0.379**			

Ct β-actin	15h control	15h A190	16h control	16h A190	17h control	17h A190
Ct β-actin	15.176	15.1182	15.0382	15.3388	15.2246	15.4253
Ct β-actin	15.1454	15.097	15.5253	15.3098	15.221	15.3916
Ct β-actin	15.1527	15.2108	15.2823	15.3835	15.3492	16.8826
Ct β-actin	15.1517	15.2276	15.3165	15.3691	15.2884	16.8404
Ct β-actin	14.7732	15.5367	15.6514	15.3818	15.3146	15.0902
Ct β-actin	15.0989	15.5332	15.5478	15.3834	15.2668	15.0827
Ct β-actin	15.5003	15.9926	15.1934	15.0416	15.9859	15.1982
Ct β-actin	15.5463	15.2258	15.0071	15.911	15.2163	15.9308
	**average**	**15.393**		**standart**	**0.4**	
